# Baseline characteristics of eyes with early residual fluid post loading phase of aflibercept therapy in neovascular AMD: PRECISE study report 3

**DOI:** 10.1038/s41433-023-02886-1

**Published:** 2023-12-15

**Authors:** Shruti Chandra, Sarega Gurudas, Ian Pearce, Martin Mckibbin, Ajay Kotagiri, Geeta Menon, Benjamin J. L. Burton, James Talks, Anna Grabowska, Faruque Ghanchi, Richard Gale, Andrea Giani, Victor Chong, Ching Ning Taffeta Chen, Luke Nicholson, Sridevi Thottarath, Swati Chandak, Sobha Sivaprasad

**Affiliations:** 1 National Institute of Health Research Moorfields Biomedical Research Centre, Moorfields Eye Hospital, London, UK; 2 Institute of Ophthalmology, University College, London, UK; 3 The Royal Liverpool and Broadgreen University Hospitals NHS Foundation Trust, Liverpool, UK; 4 Leeds Teaching Hospitals NHS Trust, Leeds, UK; 5 South Tyneside and Sunderland NHS Foundation Trust, Sunderland, UK; 6 Frimley Health NHS Foundation Trust, Surrey, UK; 7 James Paget University Hospitals NHS Foundation Trust, Norfolk, UK; 8 Newcastle Hospitals NHS Foundation Trust, Newcastle, UK; 9 King’s College Hospital NHS Foundation Trust, London, UK; 10 Bradford Teaching Hospitals NHS Foundation Trust, Bradford, UK; 11 Hull York Medical School and York, University of York and Scarborough Teaching Hospital NHS Foundation Trust, York, UK; 12 Boehringer Ingelheim, Binger Str. 173, 55216 Ingelheim am, Rhein, Germany

**Keywords:** Predictive markers, Business and industry

## Abstract

**Purpose:**

To compare the baseline characteristics in patients with and without early residual fluid (ERF) after aflibercept loading phase (LP) in patients with treatment naïve neovascular age related macular degeneration (nAMD).

**Methods:**

Patients with nAMD initiated on LP of three intravitreal aflibercept doses were recruited from December 2019 to August 2021. Baseline demographic and OCT features associated with any ERF were analysed using Generalised Estimating Equations to account for inter-eye correlation. Receiver operating characteristic (ROC) curve was performed for selection of CST threshold.

**Results:**

Of 2128 patients enrolled, 1999 eyes of 1862 patients with complete data were included. After LP, ERF was present in 1000 (50.0%), eSRF in 746(37.3%) and eIRF in 428 (21.4%) eyes. In multivariable analysis of baseline features, eyes with increased central subfield thickness (CST) (OR 1.31 per 100 microns increase [95% CI 1.22 to 1.41]; *P* < 0.001), eyes with IRF and SRF at baseline (1.62 [95% CI 1.17 to 2.22]; *P* = 0.003), and those with SRF only (OR 2.26 [95% CI 1.59 to 3.20]; *P* < 0.001) relative to IRF only were determinants of ERF. CST ≥ 418 microns had 57% sensitivity and 58% specificity to distinguish ERF from no ERF at visit 4.

**Conclusion:**

On average, 50% of eyes have ERF after aflibercept LP. Clinically relevant baseline determinants of ERF include CST ≥ 418 µ and presence of only SRF. These eyes may require further monthly treatment before extending treatment intervals.

## Introduction

The standard of treatment for macular neovascularisation (MNV) secondary to neovascular age related macular degeneration (nAMD) is intravitreal injections of anti-vascular endothelial growth factor (anti-VEGF) agents [1]. Most anti-VEGF treatment regimens are initiated with a loading phase (LP) of monthly injections for 3 doses. The macular fluid status on optical coherence tomography (OCT) at the first appointment post LP is a decision point to assess early treatment response and customize future treatment regimen for each patient [2, 3].

A proportion of eyes have early residual fluid (ERF) following LP and this outcome is previously described as ERF or ERF-free. The ERF may be either sub-retinal fluid (SRF) and/or intraretinal fluid (IRF) and are termed eSRF and eIRF and their absences are abbreviated as eSRF-free and eIRF-free respectively [3]. With the recent availability of several anti-VEGF agents, it is now timely to explore the baseline disease characteristics of eyes with ERF after LP with aflibercept, the standard comparator used in recent clinical trials on newer agents for nAMD.

Aflibercept is a recombinant fusion protein that neutralizes all VEGF-A isoforms as well as VEGF-B and placental growth factor [4]. In the pivotal trials for aflibercept in nAMD, VIEW 1 and 2 studies, ERF was found in 22.8% of eyes at 12 weeks after aflibercept LP, with 16% having eSRF and 21.3% with eIRF [5]. Recently, secondary analysis of the pooled TENAYA and LUCERNE data showed that 33% of the patients in the aflibercept arm had ERF at 12 weeks [6]. In contrast, ERF was present in 23% of the pooled faricimab cohort at 12 weeks and predominantly driven by higher resolution of SRF [7]. None of these studies report the ERF 8 weeks after LP except the HAWK and HARRIER studies, where disease activity after LP of brolucizumab was observed in 24% of patients at 16 weeks post first injection of brolucizumab compared to 34.5% in the aflibercept arm [8].

These clinical trial results indicate that although most patients with new nAMD respond rapidly to anti-VEGF treatment, ERF is seen in a proportion of patients irrespective of the agents used. These patients would likely require more aggressive treatment compared to ERF-free eyes. As clinical trials are restricted by their eligibility criteria, the proportion of patients with ERF in clinical practice and the baseline demographic and imaging factors associated with ERF is unclear.

The aim of this study was to report the differences in baseline characteristics in patients with ERF versus ERF free after aflibercept LP in patients with treatment naïve nAMD in clinical practice.

## Methods

The PRECISE study was conducted over 10 National Health Service retinal centres in the United Kingdom (UK). The aim of the study was to conduct in-depth analysis of the response of treatment naïve nAMD to LP of aflibercept therapy. The study was approved by the Institutional Review Board of National Research Service (REC number 19/LO/1385) and followed the tenets of the Declaration of Helsinki. Written informed consent was obtained from all participants before study entry (ISRCTN28276860). Recruitment period was from 18/12/2019 to 04/08/2021.

### Patient eligibility criteria

Consent to the study was obtained from patients aged 50–100 years who were initiated on intravitreal aflibercept after diagnosis of nAMD by the investigators at the local sites. Both visual acuity (VA) and Spectralis OCT scans at baseline and within 10 weeks from 3rd loading dose injection were mandatory inclusion criteria. Both eyes of an individual were recruited if eligible. Exclusion Criteria included co-existent ocular disease that, in the opinion of the investigator, might affect or alter visual acuity during the study, poor image quality and missing scans.

### Data collection

Demographic data collected included age, gender and ethnicity. Eye level data included VA and OCT scans done at baseline and within 10 weeks after 3rd injection. Anonymised data were entered in a web-based database (Playon Ltd, Bengaluru, India). Routine raster scan protocol centred at the fovea (range 20°x25° to 30°x20°) in the Heidelberg Engineering Systems “Spectralis HRA + OCT” or “Spectralis OCT” systems. The line scan protocols on Heidelberg Spectralis scans ranged from 19 to 49 (19, 25, 31 or 49). Retinal images were anonymised and transferred by encrypted USB to Moorfields Eye Hospital for grading.

### OCT grading protocol

A pre-defined OCT grading manual was used to grade the OCT scans. The images were graded by five Medical Retina Fellows after grading on a test set of 50 OCT images. Each scan was corrected for any segmentation errors and foveal centration. The grading results were recorded on standardised case report forms. The ERF was defined as presence of any eIRF or eSRF anywhere in the 6×6 mm macula scan at the first clinic appointment after LP. The final visit was up to 140 days after the first injection were permitted. The CST was measured from the internal limiting membrane (ILM) to the Bruch’s membrane (BrM). The type of neovascularization was graded on OCT as per the Consensus on Neovascular Age-Related Macular Degeneration Nomenclature Study Group (CONAN) classification [9].

#### Outcomes

The primary outcome of the study was the presence of ERF at the final visit. Other outcomes included the presence of eSRF or eIRF at the final visit.

### Statistical analysis

Data were summarised with mean ± SD or median (interquartile range [IQR]) for normally and non-normally distributed continuous variables, respectively and *n* (%) for categorical variables. Univariate and multivariable associations between demography and OCT features and the binary outcomes for ERF, eSRF and eIRF were reported using Odds Ratio (95% CI) and *P*-value. Generalised Estimating Equations (GEE) with an exchangeable working correlation structure were used to account for the within-participant correlation among those with data from both eyes [10, 11]. Black, south Asians, other Asians and other ethnicities were categorised as Non-White in models due to insufficient sample size. CST was analysed as a continuous variable and in quartiles, ranging from low CST (quartile 1) to high CST (quartile 4). Receiver operating characteristic (ROC) curve analysis was performed for CST and IRF ± SRF using clustered bootstrap with 1000 replicates to estimate the bias corrected 95% confidence intervals for the area under the curve (AUC), and optimal thresholds for CST were selected based on maximising Youden’s index, defined as sensitivity + specificity – 1 [12, 13]. Bootstrapping allows for probability-based inference for the AUC and corrects for the inter-eye correlation with respect to the estimation of the standard error of the estimated AUC. Sensitivity analysis was performed to assess associations of MNV features with the study outcomes, excluding patients that attended their final visit less than 104 days from their first aflibercept injection. Statistical analysis was undertaken using R version 4.1.2 statistical software package and Stata MP 15.

## Results

A total of 2039 gradable eyes of 1901 patients were initiated on aflibercept therapy for nAMD. The flow chart shows how 1999 eyes of 1862 patients was derived to form the sample for analysis (Fig. S1).

Table 1 shows the age wise distribution, vision and some of the imaging characteristics of the eyes included in the study. Table S1 shows the detailed demographic, clinical and ocular characteristics. Mean age was 79.3 (SD 7.8) years, 1126 (60.5%) were women and 1772 (95.2%) participants were white. The median time interval between the first aflibercept injection and the final visit was 112 days (IQR 98 to 119 days) in the total cohort, and modes located at 86.7 (~12 weeks) and 114 days (~16 weeks). CST was categorized as less than or equal to 340 µm in quartile 1 (Q1), 341 µm to 415 µm in quartile 2 (Q2), 416 µm to 525 µm in quartile 3 (Q3) and greater than 525 µm in quartile 4 (Q4). Median CST was 416 µm (IQR 340 µm to 526 µm).

**Table 1:** Demography, baseline clinical and ocular characteristics overall and by fluid status/type.

Variable	Overall, *N* = 1999 eyes of 1862 patients	Visit 4 ^a^					
		ERF status		eSRF status		eIRF status	
		No ERF, *N* = 999 eyes of 950 patients	ERF, *N* = 1000 eyes of 973 patients	No eSRF, *N* = 1253 eyes of 1174 patients	eSRF, *N* = 746 eyes of 734 patients	No eIRF, *N* = 1571 eyes of 1481 patients	eIRF, *N* = 428 eyes of 417 patients
Patient level (*N* = 1862) Age, years	79.3 (7.8)	80.4 (7.7)	78.3 (7.6)	80.4(7.7)	77.7 (7.5)	79.3 (7.9)	79.8 (7.3)
Eye level (*N* = 1999 eyes)							
Mean Baseline visual acuity, ETDRS letters	58.0 (14.5)	57.9 (14.3)	58.1 (14.9)	57.0 (14.5)	59.5 (14.6)	59.1 (14.1)	53.5 (15.6)
Baseline visual acuity categories, ETDRS letters							
<54	648 (32.4%)	318 (31.8%)	330 (33.0%)	433 (34.6%)	215 (28.8%)	449 (28.6%)	199 (46.5%)
54–67	715 (35.8%)	373 (37.3%)	342 (34.2%)	459 (36.6%)	256 (34.3%)	573 (36.5%)	142 (33.2%)
≥68	636 (31.8%)	308 (30.8%)	328 (32.8%)	361 (28.8%)	275 (36.9%)	549 (34.9%)	87 (20.3%)
Central subfield thickness in microns	416 (340, 526)	393 (322, 492)	437 (362, 567)	401 (326, 509)	436 (364, 567)	404 (334, 510)	467 (366, 596)
Presence of Atrophy	421 (21.1%)	265 (26.5%)	156 (15.6%)	341 (27.2%)	80 (10.7%)	314 (20.0%)	107 (25.0%)
Presence of fibrosis	292 (14.6%)	128 (12.8%)	164 (16.4%)	189 (15.1%)	103 (13.8%)	183 (11.6%)	109 (25.5%)
Presence of SHRM	1164 (58.2%)	576 (57.7%)	588 (58.8%)	722 (57.6%)	442 (59.2%)	896 (57.0%)	268 (62.6%)
Presence of SDD	598 (29.9%)	337 (33.7%)	261 (26.1%)	429 (34.2%)	169 (22.7%)	466 (29.7%)	132 (30.8%)
Presence of VMT or ERM	251 (12.6%)	125 (12.5%)	126 (12.6%)	179 (14.3%)	72 (9.7%)	176 (11.2%)	75 (17.5%)
EZ and ELM combination							
Intact EZ and ELM	847 (42.4%)	378 (37.8%)	469 (46.9%)	451 (36.0%)	396 (53.1%)	736 (46.8%)	111 (25.9%)
Either EZ or ELM loss	584 (29.2%)	313 (31.3%)	271 (27.1%)	427 (34.1%)	157 (21.0%)	401 (25.5%)	183 (42.8%)
Both ungradable	568 (28.4%)	308 (30.8%)	260 (26.0%)	375 (29.9%)	193 (25.9%)	434 (27.6%)	134 (31.3%)

*CONAN* consensus on neovascular AMD nomenclature, *eIRF* early intraretinal fluid, *ERF* early residual fluid, *ERM* epiretinal membrane, *eSRF* early subretinal fluid, *ETDRS* early treatment diabetic retinopathy study, *ELM* external limiting membrane, *EZ* ellipsoid zone, *HRF* hyperreflective foci, *IQR* interquartile range, *IRF* intraretinal fluid, *MNV* macular neovascularization, *OCT* optical coherence tomography, *OR* odds ratio, ORT outer retinal tubulation, *PCV* polypoidal vasculopathy, *PED* pigment epithelial detachment, *RAP* retinal angiomatous proliferation, *SD* standard deviation, *SDD* subretinal drusenoid deposits, *SHRM* subretinal hyperreflective material, *SRF* subretinal fluid, *VA* visual acuity, *VMT* vitreomacular traction.

^a^The same patients may be present in different residual macular fluid groups, hence the number of patients may not sum up to the total of 1862 patients.

### Baseline macular fluid status

At baseline, a third of eyes (672 of 1999 [33.6%]) presented with both IRF and SRF. More than 80% had SRF (1654 of 1999 [82.7%]) and approximately half of the eyes had IRF (1017 of 1999 [50.9%]). A total of 982 (49.1%) had SRF without IRF at presentation.

### Early residual fluid (ERF)

**Fig. 1:**
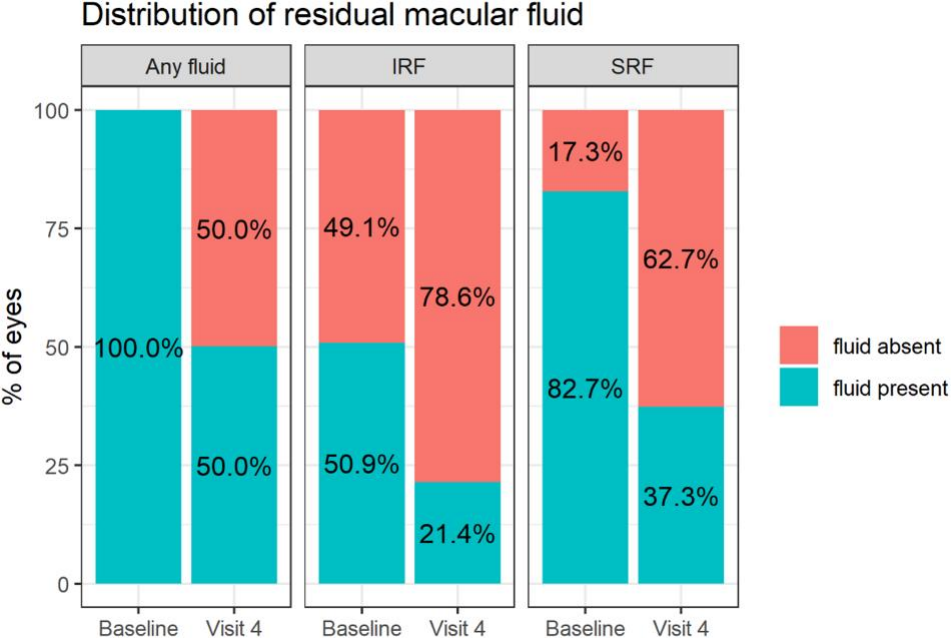
Distribution of fluid at baseline and post-loading. IRF intraretinal fluid, SRF subretinal fluid.

After LP, ERF was present in 1000 eyes (50.0%, Fig. 1). The proportion with eSRF and eIRF were 37.3% (*N* = 746) and 21.4% (*N* = 428) respectively. The proportion of eyes with co-existent eSRF and eIRF was 8.7% (*N* = 174 eyes), and 50.0% of total cohort had no macular fluid (*N* = 999 eyes). Table 2 shows the baseline and post LP VA (unadjusted and adjusted) based on the distribution of ERF. Eyes with residual eIRF had the worst VA across all groups.

**Table 2:** Presenting and post-loading VA based on distribution of ERF at visit 4.

ERF at visit 4	Baseline Mean VA (SD)	Post-loading (Visit 4)	
		Mean VA (SD)	Adjusted Mean VA (SE)^a^
**ERF**	58.1 (SD 14.8)	61.3 (16.5)	61.7 (SE 0.35)
**No ERF**	57.9 (SD 14.3)	64.4 (16.0)	63.4 (SE 0.36)
**eSRF (**±**eIRF)**	59.5 (SD 14.5)	63.2 (13.9)	62.1 (SE 0.41)
**eSRF only**	61.5 (SD 13.4)	65.2 (12.8)	62.7 (SE 0.45)
**No eSRF**	57.1 (SD 14.5)	62.1 (16.0)	62.8 (SE 0.32)
**eIRF (**±**eSRF)**	53.6 (SD 15.5)	57.2 (16.4)	60.3 (SE 0.56)
**eIRF only**	54.0 (SD 15.0)	57.6 (17.0)	60.4 (SE 0.71)
**No eIRF**	59.2 (SD 14.0)	64.0 (14.6)	63.2 (SE 0.29)

*ERF* early residual fluid, *eSRF* early subretinal fluid, *eIRF* early intraretinal fluid.

^a^GEE model with visit 4 visual acuity as the outcome and adjusted for baseline visual acuity.

### Associations of ERF and its distribution

**Fig. 2:**
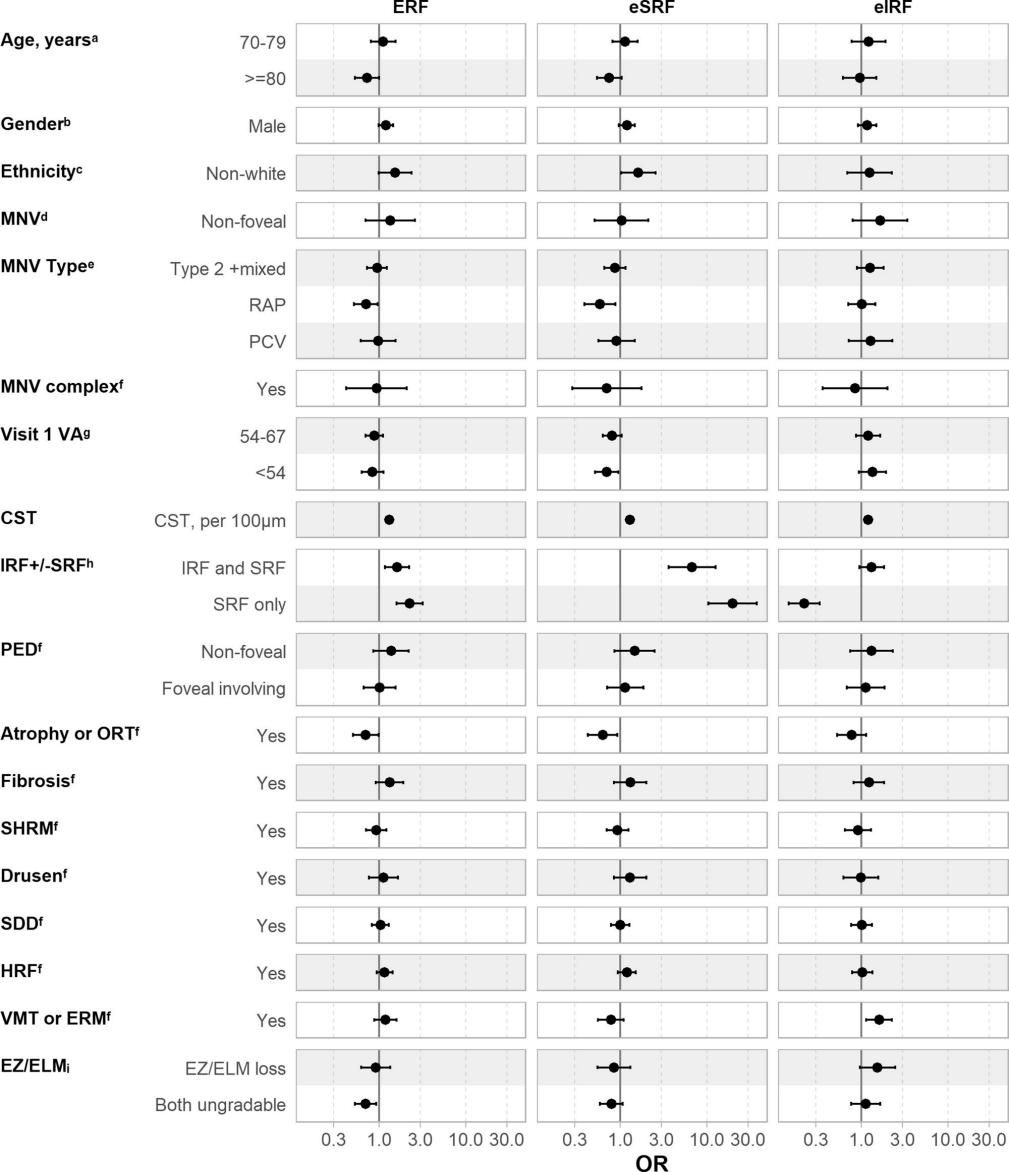
Plot of Odds Ratio with 95% CIs showing baseline demographic and OCT characteristics associated with eRF, eSRF and eIRF at visit 4 – multivariable analysis using Generalised Estimating Equations (GEE). Non-White includes Black, South Asian, other Asian or other ethnic categories, CI confidence interval, CONAN consensus on neovascular AMD nomenclature, eIRF early intraretinal fluid, eRF early residual fluid, ERM epiretinal membrane, eSRF early subretinal fluid, ETDRS early treatment diabetic retinopathy study, ELM external limiting membrane, EZ ellipsoid zone, GEE generalized estimating equation, HRF hyperreflective foci, IQR interquartile range, IRF intraretinal fluid, MNV macular neovascularization, OCT optical coherence tomography, OR Odds Ratio, ORT outer retinal tubulation, PCV polypoidal vasculopathy, PED pigment epithelial detachment, RAP retinal angiomatous proliferation, SD standard deviation, SDD subretinal drusenoid deposits, SHRM subretinal hyperreflective material, SRF subretinal fluid, VA visual acuity, VMT vitreomacular traction. The ratio axis is displayed on the logarithmic scale to provide a visual description of the uncertainty associated with each estimate. Reference categories were: ^a^<70 years, ^b^Female, ^c^White, ^d^MNV foveal involving, ^e^Type 1, ^f^Absent or No, ^g^VA ≥ 68 ETDRS letters,^h^IRF only, ^i^EZ and ELM intact.

Univariate and multivariable analysis of participant demographic and OCT characteristics were performed to study its associations with presence of ERF, eSRF and eIRF (Table S2 and Fig. 2).

Variables with increased odds of ERF in multivariable analysis included; presence of IRF and SRF, SRF only and increased CST. Variables associated with a reduction of odds of ERF included participants aged 80 years and above, eyes with retinal angiomatous proliferation (RAP) relative to Type 1, presence of atrophy or outer retinal tubulation, and ungradable ellipsoid zone (EZ) and external limiting membrane (ELM).

Non-whites, increased CST, presence of IRF and SRF and presence of SRF only were associated with increased odds of eSRF. While visual acuity <54 ETDRS letters, RAP lesions, presence of atrophy or outer retinal tubulation were associated with reduced odds of SRF at visit 4.

Variables that showed increased odds for IRF at visit 4 only included increased CST and presence of vitreomacular traction or ERM. SRF only (without IRF) at baseline was associated with reduced odds of eIRF.

Sensitivity analysis excluding 592 eyes where final visit occurred less than 104 days from the first aflibercept injection showed that our results remain robust to the main analysis (Table S3).

### Optimal threshold for CST

**Fig. 3:**
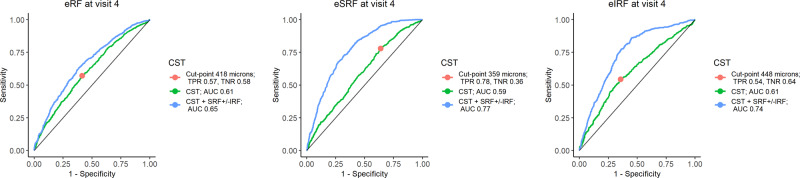
The receiver operating characteristic (ROC) curves for CST in detecting eRF, eSRF and eIRF at visit 4. CST central subfield thickness, AUC area under the curve, TPR true positive rate (Sensitivity), TNR true negative rate (Specificity), eRF early residual fluid, eSRF early subretinal fluid, eIRF early intraretinal fluid. Optimal threshold points that maximize Youden’s index indicated in red. AUC and bias corrected 95% confidence intervals for CST were 0.61 (95% CI 0.58–0.63) for eRF, 0.59 (95% CI 0.56–0.62) for eSRF and 0.61 (95% CI 0.58–0.64) for eIRF. AUCs and bias corrected 95% confidence intervals for CST and IRF ± SRF were 0.65 (95% bias-corrected CI 0.64–0.68) for eRF, 0.77 (95% bias-corrected CI 0.75–0.79) for eSRF and 0.74 (95% bias-corrected CI 0.72–0.77) for eIRF.

ROC analysis was performed to find the optimal cut-off point for CST capable of discriminating presence of ERF, eSRF and eIRF at visit 4 (Fig. 3). AUCs were comparable and ranged from 0.59 to 0.61 for distinguishing presence and absence of ERF, eSRF and eIRF using CST alone. CST ≥ 418 microns had 57% sensitivity and 58% specificity to distinguish ERF from no ERF at visit 4. While CST ≥ 359 microns was able to distinguish eSRF from no eSRF at visit 4 with 78 and 36% sensitivity and specificity respectively. CST ≥ 448 microns identified eIRF with 54% sensitivity and confirmed no eIRF with 64% specificity. CST together with SRF and/or IRF (IRF only, SRF and IRF, SRF only) yielded AUCs 0.65 (95% bias-corrected CI 0.64–0.68), 0.77 (95% bias-corrected CI 0.75–0.79) and 0.74 (95% bias-corrected CI 0.72–0.77) for predicting eRF, eSRF and eIRF respectively.

## Discussion

In this study, we report the proportion of patients with ERF, eSRF, eIRF after aflibercept LP for nAMD and their determinants at baseline.

Fifty percent of the cohort had ERF after LP and this proportion is similar to that achieved by the aflibercept cohort at 8 weeks post loading injections in TENAYA and LUCERNE. When we consider the distribution of ERF, the proportion of patients with no IRF (with or without SRF) was 78.6% and SRF with or without IRF was 62.7% in our study. These results also mirror those obtained 8 weeks post loading in the aflibercept arm of TENAYA and LUCERNE (76% with no IRF and 62% with no SRF) [6].

Baseline factors associated with ERF included non-white ethnicity, males, increased CST, eyes presenting with IRF and SRF or with SRF only. These results indicate that patients with these features are likely to require further monthly injections for complete fluid resolution.

A key observation is that high baseline CST is associated with higher prevalence of ERF. Similar observations were noted in the VIEW 1 and 2 studies [5]. Post-hoc exploration of baseline features in the ALTAIR study also showed that patients with increased central retinal thickness, high PED height and absence of PCV or subretinal haemorrhage were more likely to have retinal fluid at week 16 [14]. Secondary analysis of the combined faricimab arms in TENAYA and LUCERNE also showed that eyes with increased CST at baseline were more likely to require 8 weekly dosing compared to the other two cohorts dosed at extended intervals [15]. Similar results were also observed in HAWK and HARRIER analysis [8]. These findings highlight that irrespective of the drug used, eyes presenting with high CST are the ‘difficult to treat’ group and are likely to require frequent injections. Therefore, these eyes necessitate further monthly injections before considering extension of treatment intervals. In this study, a CST of ≥ 418 microns is likely associated with ERF.

Although non-white ethnicity was associated with ERF and eSRF, only 5% of the study population were of non-white origin. Therefore, a further study comparing similar proportions of white and non-white patients is required to validate these findings.

Patients aged 80 years or older were less likely to have ERF. This may be partly explained by the higher prevalence of RAP and atrophy in older individuals, which were both also associated with decreased odds of having ERF in this study. This finding is in keeping with the secondary analysis of TENAYA and LUCERNE, which also demonstrated higher proportions of RAPs in the eyes that were maintained in the Q16W arm [15].

When we consider the relationship of VA in eyes with ERF and its distribution, the findings in our real-life study are comparable to the results of a post-hoc analysis of aflibercept LP in the ARIES study [16, 17]. Both the presenting VA and the VA outcome adjusted for baseline VA and other potential confounders were numerically higher in eyes with eSRF. The converse was true for eIRF. The negative impact of IRF has been established in previous reports at various time-points [14, 18–21]. Our results that high CST and poorer VA at baseline are associated with eIRF indicate that these eyes also need to be treated more aggressively.

Interestingly, baseline atrophy was associated with less odds of both ERF and eSRF. The complete loss of the RPE barrier may indeed facilitate unimpeded passage of aflibercept to the MNV. This may also explain why eyes with ungradable ellipsoid layer are less likely to have ERF and SRF post-loading. In active MNV eyes, features like subretinal fluid or haemorrhage or subretinal hyperreflective material make it challenging to grade ellipsoid layer due to significant back shadowing or blurring of outer layer details. As such any underlying EZ/ELM loss indicating atrophy might be missed at baseline. On the contrary, baseline atrophy did not influence the resolution of IRF.

Our study has the following strengths. This study analyzed a large real-world data of nearly 2000 eyes collected from 10 centers in UK, thereby representative of clinical practices across various centers. In addition, the data is uniform with all receiving three loading doses of aflibercept for treatment naïve neovascular AMD eyes and all imaging was on Heidelberg, Spectralis SD-OCT. All eyes underwent exhaustive and meticulous grading after manual correction of segmentation, if required. Presence of specific imaging biomarkers that could potentially determine residual macular fluid, including but not limited to SHRM, ORT, VMT, ERM, loss of ELM and EZ were graded by trained graders with excellent inter-grader agreement. As both eyes of some patients were included in the study robust analytical tools like generalized estimating equations were employed to account for any effect. The analyses were also adjusted for VA and time to follow-up to ensure strength of results. Finally, this study provides unique data on baseline determinants of ERF in real-life. However, there are some limitations to this study. The classification of MNV subtypes is OCT based CONAN classification and we did not have concurrent fluorescein or indocyanine green angiography to confirm the MNV subtype [9].

In conclusion, ERF is present in 50% of individuals treated with aflibercept LP. The baseline characteristics, including non-white ethnicity, males, increased CST (418 microns or more), eyes presenting with IRF and SRF or with SRF are at risk of developing ERF. These eyes are likely to require more monthly injections to attain stability before extension of treatment intervals are planned.

## Summary

### What was known before

- Aflibercept is the most commonly used first-line agent for treatment of naive neovascular AMD (nAMD) in the UK. Aflibercept is also the standard comparator used in recent clinical trials on newer agents for nAMD. A proportion of eyes have early residual fluid (ERF) following LP and this outcome is previously described as ERF or ERF-free. As clinical trials are restricted by their eligibility criteria, the proportion of patients with ERF in clinical practice and the baseline demographic and imaging factors associated with ERF is unclear.

### What this study adds

- Fifty percent of the cohort had ERF after LP in the real world setting. This proportion is similar to that achieved by the aflibercept cohort across various clinical trials. Baseline factors associated with ERF included non-white ethnicity, males, increased CST, eyes presenting with IRF and SRF or with SRF only. These results indicate that patients with these features are likely to require further monthly injections for complete fluid resolution. Key observation is eyes with CST > 418 and SRF only at baseline are clinically relevant markers that determine ERF post loading.

### Supplementary information

Figure S1

Table S1

Table S2

Table S3

## Data Availability

The data that support the findings of this study are not openly available due to reasons of sensitivity and are available from the corresponding author upon reasonable request.
