# Supplementary material for: Baseline characteristics of eyes with early residual fluid post loading phase of aflibercept therapy in neovascular AMD: PRECISE study report 3
Source: Eye (Lond). 2023 Dec 15;38(7):1301–7. doi: 10.1038/s41433-023-02886-1 (PMC11076629; doi:10.1038/s41433-023-02886-1)
Supplement: Supplementary file 1 — Figure S1 [file 41433_2023_2886_MOESM1_ESM.docx]

Numbers recruited across all 10 PRECISE sites and entered onto the database

Inclusion Criteria:

- Treatment naïve wet AMD eye in study eye
- Received three aflibercept loading doses

**(N = 2274 eyes of 2128 patients )**

# Enrollment

Excluded **(n = 235 eyes of 227 patients)**

Exclusion Criteria:

1. Scans not sent to reading center - 107
2. Mandatory visit (V1/V4) scans missing-52
3. Wrong diagnosis-33

*(No CNV-19; PEVAC-1; Mactel-1; Myopic CNV-4; RVO-2; PAMM-2; RAM-1; AOFVD-1; Angioid streak CNV-1; CSCR CNV-1)*

1. Ungradable-16

*(Poor quality (</=20dB) -8; >500µ foveal atrophy-1; Subfoveal Haemorrhage>50% of lesion-3; Central fibrosis>1000µ-4)*

1. Outside visit window-23
2. Withdrawal from study – 4

#

**N = 2,039 eyes of 1,901 patients**

**Follow up**

Excluded for Report 3 Analysis **(n= 40 eyes of 39 patients)**

Reasons for exclusion:

1. Final follow up beyond 140 days of baseline – 37 eyes
2. Only subretinal hyperreflective material at baseline, no fluid – 3 eyes

# Analysis

**Final Analyzed Cohort**

**N = 1,999 eyes of 1,862 patients**

Figure S1: Participant Flow

***Abbreviations**: AMD- Age related macular degeneration; Angioid streak CNV- Angioid streak related choroidal neovascularization; AOFVD- Adult onset foveomacular vitelliform dystrophy; CSCR CNV- Central serous chorioretinopathy related choroidal neovascularization; CNV-Choroidal neovascularization; dB- decibel; Mactel- Macular telangiectasia; Myopic CNV- Myopic choroidal neovascularization; PAMM- Paracentral acute middle maculopathy; PEVAC-Perifoveal exudative vascular anomalous complex; RAM- Retinal artery macroaneurysm; RVO- Retinal vein occlusion; V1- Visit 1; V4- Visit 4; µ-microns
