# Supplementary material for: Baseline characteristics of eyes with early residual fluid post loading phase of aflibercept therapy in neovascular AMD: PRECISE study report 3
Source: Eye (Lond). 2023 Dec 15;38(7):1301–7. doi: 10.1038/s41433-023-02886-1 (PMC11076629; doi:10.1038/s41433-023-02886-1)
Supplement: Supplementary file 2 — Table S1 [file 41433_2023_2886_MOESM2_ESM.docx]

**Table S1: Demography, baseline clinical and ocular characteristics overall and by fluid status/type**

| **Variable** | **Overall**, N = 1,999 eyes of 1,862 patients | **Visit 4 ^a^** | | | | | |
| --- | --- | --- | --- | --- | --- | --- | --- |
|  |  | **ERF status** | | **eSRF status** | | **eIRF status** | |
|  |  | No ERF, N = 999 eyes of 950 patients | ERF, N = 1,000 eyes of 973 patients | No eSRF, N = 1,253 eyes of 1,174 patients | eSRF, N = 746 eyes of 734 patients | No eIRF, N = 1,571 eyes of 1,481 patients | eIRF, N = 428 eyes of 417 patients |
| **Patient level (N=1,862)** |  |  |  |  |  |  |  |
| **Age, years** | 79.3 (7.8) | 80.4 (7.7) | 78.3 (7.6) | 80.4(7.7) | 77.7 (7.5) | 79.3 (7.9) | 79.8 (7.3) |
| **Age, years** |  |  |  |  |  |  |  |
| < 70 | 198 (10.6%) | 88 (9.3%) | 116 (11.9%) | 108 (9.2%) | 96 (13.1%) | 164 (11.1%) | 36 (8.6%) |
| 70-79 | 662 (35.6%) | 286 (30.1%) | 398 (40.9%) | 360 (30.7%) | 319 (43.5%) | 526 (35.5%) | 150 (36.0%) |
| >=80 | 1002 (53.8%) | 576 (60.6%) | 459 (47.2%) | 706 (60.1%) | 319 (43.5%) | 791 (53.4%) | 231 (55.4%) |
| **Gender** |  |  |  |  |  |  |  |
| Female | 1126 (60.5%) | 606 (63.8%) | 560 (57.6%) | 739 (62.9%) | 415 (56.5%) | 904 (61.0%) | 243 (58.3%) |
| Male | 736 (39.5%) | 344 (36.2%) | 413 (42.4%) | 435 (37.1%) | 319 (43.5%) | 577 (39.0%) | 174 (41.7%) |
| **Ethnicity** |  |  |  |  |  |  |  |
| Black | 11 (0.6%) | 5 (0.5%) | 6 (0.6%) | 6 (0.5%) | 5 (0.7%) | 10 (0.7%) | 1 (0.2%) |
| Other | 54 (2.9%) | 22 (2.3%) | 34 (3.5%) | 28 (2.4%) | 28 (3.8%) | 42 (2.8%) | 13 (3.1%) |
| Other Asian | 9 (0.5%) | 3 (0.3%) | 6 (0.6%) | 4 (0.3%) | 5 (0.7%) | 6 (0.4%) | 3 (0.7%) |
| South Asian | 16 (0.9%) | 8 (0.8%) | 8 (0.8%) | 11 (0.9%) | 5 (0.7%) | 12 (0.8%) | 4 (1.0%) |
| White | 1772 (95.2%) | 912 (96.0%) | 919 (94.5%) | 1125 (95.8%) | 691 (94.1%) | 1411 (95.3%) | 396 (95.0%) |
| **Number of patients with 2 eyes** | 137 (6.9%) | 49 (4.9%) | 27 (2.7%) | 79 (6.3%) | 12 (1.6%) | 90 (5.7%) | 11 (2.6%) |
| **Eye level (N=1,999 eyes)** |  |  |  |  |  |  |  |
| **Time since first aflibercept injection** | 112 (98, 119) | 112 (91, 118) | 113 (110, 119) | 112 (93, 118) | 114 (112, 120) | 112 (96, 119) | 113 (112, 119) |
| **Visit 1 visual acuity, ETDRS** | 58.0 (14.5) | 57.9 (14.3) | 58.1 (14.9) | 57.0 (14.5) | 59.5 (14.6) | 59.1 (14.1) | 53.5 (15.6) |
| **Visit 1 visual acuity categories, ETDRS** |  |  |  |  |  |  |  |
| <54 | 648 (32.4%) | 318 (31.8%) | 330 (33.0%) | 433 (34.6%) | 215 (28.8%) | 449 (28.6%) | 199 (46.5%) |
| 54-67 | 715 (35.8%) | 373 (37.3%) | 342 (34.2%) | 459 (36.6%) | 256 (34.3%) | 573 (36.5%) | 142 (33.2%) |
| >=68 | 636 (31.8%) | 308 (30.8%) | 328 (32.8%) | 361 (28.8%) | 275 (36.9%) | 549 (34.9%) | 87 (20.3%) |
| **Presence of MNV** |  |  |  |  |  |  |  |
| Yes, Foveal involving | 1,914 (95.7%) | 955 (95.6%) | 959 (95.9%) | 1,196 (95.5%) | 718 (96.2%) | 1,506 (95.9%) | 408 (95.3%) |
| Yes, Non-Foveal | 85 (4.3%) | 44 (4.4%) | 41 (4.1%) | 57 (4.5%) | 28 (3.8%) | 65 (4.1%) | 20 (4.7%) |
| **MNV Type based on OCT CONAN criteria** |  |  |  |  |  |  |  |
| Type 1 | 781 (39.1%) | 346 (34.6%) | 435 (43.5%) | 403 (32.2%) | 378 (50.7%) | 672 (42.8%) | 109 (25.5%) |
| Type 2 | 652 (32.6%) | 314 (31.4%) | 338 (33.8%) | 396 (31.6%) | 256 (34.3%) | 500 (31.8%) | 152 (35.5%) |
| RAP | 440 (22.0%) | 291 (29.1%) | 149 (14.9%) | 390 (31.1%) | 50 (6.7%) | 307 (19.5%) | 133 (31.1%) |
| PCV | 126 (6.3%) | 48 (4.8%) | 78 (7.8%) | 64 (5.1%) | 62 (8.3%) | 92 (5.9%) | 34 (7.9%) |
| **Presence of any component of MNV complex** | 1,945 (97.3%) | 971 (97.2%) | 974 (97.4%) | 1,218 (97.2%) | 727 (97.5%) | 1,530 (97.4%) | 415 (97.0%) |
| **Central subfield thickness in microns** | 416 (340, 526) | 393 (322, 492) | 437 (362, 567) | 401 (326, 509) | 436 (364, 567) | 404 (334, 510) | 467 (366, 596) |
| **Central subfield thickness quartiles in microns** |  |  |  |  |  |  |  |
| [137-340] | 503 (25.2%) | 313 (31.3%) | 190 (19.0%) | 370 (29.5%) | 133 (17.8%) | 426 (27.1%) | 77 (18.0%) |
| (340,415] | 496 (24.8%) | 261 (26.1%) | 235 (23.5%) | 313 (25.0%) | 183 (24.5%) | 412 (26.2%) | 84 (19.6%) |
| (415,525] | 499 (25.0%) | 233 (23.3%) | 266 (26.6%) | 295 (23.5%) | 204 (27.3%) | 390 (24.8%) | 109 (25.5%) |
| (525, 1423] | 501 (25.1%) | 192 (19.2%) | 309 (30.9%) | 275 (21.9%) | 226 (30.3%) | 343 (21.8%) | 158 (36.9%) |
| **Presence of IRF** | 1,017 (50.9%) | 594 (59.5%) | 423 (42.3%) | 822 (65.6%) | 195 (26.1%) | 668 (42.5%) | 349 (81.5%) |
| **Presence of SRF** | 1,654 (82.7%) | 753 (75.4%) | 901 (90.1%) | 920 (73.4%) | 734 (98.4%) | 1,318 (83.9%) | 336 (78.5%) |
| **Distribution of macular fluid** |  |  |  |  |  |  |  |
| SRF and IRF | 672 (33.6%) | 348 (34.8%) | 324 (32.4%) | 489 (39.0%) | 183 (24.5%) | 415 (26.4%) | 257 (60.0%) |
| SRF only | 982 (49.1%) | 405 (40.5%) | 577 (57.7%) | 431 (34.4%) | 551 (73.9%) | 903 (57.5%) | 79 (18.5%) |
| IRF only | 345 (17.3%) | 246 (24.6%) | 99 (9.9%) | 333 (26.6%) | 12 (1.6%) | 253 (16.1%) | 92 (21.5%) |
| **Presence of PED** |  |  |  |  |  |  |  |
| No | 101 (5.1%) | 53 (5.3%) | 48 (4.8%) | 70 (5.6%) | 31 (4.2%) | 76 (4.8%) | 25 (5.8%) |
| Yes, Foveal involving | 1,558 (77.9%) | 774 (77.5%) | 784 (78.4%) | 958 (76.5%) | 600 (80.4%) | 1,234 (78.5%) | 324 (75.7%) |
| Yes, Non-Foveal | 340 (17.0%) | 172 (17.2%) | 168 (16.8%) | 225 (18.0%) | 115 (15.4%) | 261 (16.6%) | 79 (18.5%) |
| **Presence of Atrophy** | 421 (21.1%) | 265 (26.5%) | 156 (15.6%) | 341 (27.2%) | 80 (10.7%) | 314 (20.0%) | 107 (25.0%) |
| **Presence of fibrosis** | 292 (14.6%) | 128 (12.8%) | 164 (16.4%) | 189 (15.1%) | 103 (13.8%) | 183 (11.6%) | 109 (25.5%) |
| **Presence of SHRM** | 1,164 (58.2%) | 576 (57.7%) | 588 (58.8%) | 722 (57.6%) | 442 (59.2%) | 896 (57.0%) | 268 (62.6%) |
| **Presence of ORT** | 51 (2.6%) | 31 (3.1%) | 20 (2.0%) | 39 (3.1%) | 12 (1.6%) | 38 (2.4%) | 13 (3.0%) |
| **Presence of Drusen** | 1,833 (91.7%) | 920 (92.1%) | 913 (91.3%) | 1,148 (91.6%) | 685 (91.8%) | 1,448 (92.2%) | 385 (90.0%) |
| **Presence of SDD** | 598 (29.9%) | 337 (33.7%) | 261 (26.1%) | 429 (34.2%) | 169 (22.7%) | 466 (29.7%) | 132 (30.8%) |
| **Presence of HRF** | 1,401 (70.1%) | 697 (69.8%) | 704 (70.4%) | 883 (70.5%) | 518 (69.4%) | 1,082 (68.9%) | 319 (74.5%) |
| **Presence of VMT** | 39 (2.0%) | 13 (1.3%) | 26 (2.6%) | 25 (2.0%) | 14 (1.9%) | 24 (1.5%) | 15 (3.5%) |
| **Presence of ERM** | 217 (10.9%) | 114 (11.4%) | 103 (10.3%) | 158 (12.6%) | 59 (7.9%) | 155 (9.9%) | 62 (14.5%) |
| **Presence of VMT or ERM** | 251 (12.6%) | 125 (12.5%) | 126 (12.6%) | 179 (14.3%) | 72 (9.7%) | 176 (11.2%) | 75 (17.5%) |
| **Loss of Ellipsoid zone** |  |  |  |  |  |  |  |
| No | 578 (28.9%) | 267 (26.7%) | 311 (31.1%) | 315 (25.1%) | 263 (35.3%) | 506 (32.2%) | 72 (16.8%) |
| Yes | 584 (29.2%) | 313 (31.3%) | 271 (27.1%) | 427 (34.1%) | 157 (21.0%) | 401 (25.5%) | 183 (42.8%) |
| Not gradeable | 837 (41.9%) | 419 (41.9%) | 418 (41.8%) | 511 (40.8%) | 326 (43.7%) | 664 (42.3%) | 173 (40.4%) |
| **Loss of External limiting membrane** |  |  |  |  |  |  |  |
| No | 883 (44.2%) | 394 (39.4%) | 489 (48.9%) | 471 (37.6%) | 412 (55.2%) | 764 (48.6%) | 119 (27.8%) |
| Yes | 536 (26.8%) | 291 (29.1%) | 245 (24.5%) | 398 (31.8%) | 138 (18.5%) | 364 (23.2%) | 172 (40.2%) |
| Not gradeable | 580 (29.0%) | 314 (31.4%) | 266 (26.6%) | 384 (30.6%) | 196 (26.3%) | 443 (28.2%) | 137 (32.0%) |
| **EZ and ELM combination** |  |  |  |  |  |  |  |
| Intact EZ and ELM | 847 (42.4%) | 378 (37.8%) | 469 (46.9%) | 451 (36.0%) | 396 (53.1%) | 736 (46.8%) | 111 (25.9%) |
| Either EZ orELM loss | 584 (29.2%) | 313 (31.3%) | 271 (27.1%) | 427 (34.1%) | 157 (21.0%) | 401 (25.5%) | 183 (42.8%) |
| Both ungradable | 568 (28.4%) | 308 (30.8%) | 260 (26.0%) | 375 (29.9%) | 193 (25.9%) | 434 (27.6%) | 134 (31.3%) |

*Abbreviations: CONAN-Consensus on*[*Neovascular AMD*](https://www.sciencedirect.com/topics/medicine-and-dentistry/wet-macular-degeneration)*Nomenclature; eIRF – Early intraretinal fluid; ERF – Early residual fluid; ERM- Epiretinal membrane; eSRF – Early subretinal fluid; ETDRS – Early treatment diabetic retinopathy study; ELM- External limiting membrane; EZ- Ellipsoid zone; HRF – Hyperreflective foci; IQR- interquartile range; IRF- Intraretinal fluid; MNV – Macular neovascularisation; OCT – Optical Coherence Tomography; OR- Odds Ratio; ORT – Outer retinal tubulation; PCV-polypoidal vasculopathy; PED – Pigment Epithelial Detachment; RAP-retinal angiomatous proliferation; SD-standard deviation; SDD- Subretinal drusenoid deposits; SHRM- Subretinal hyperreflective material; SRF- Subretinal fluid; VA- Visual Acuity; VMT- Vitreomacular traction.*

*^a^ The same patients may be present in different residual macular fluid groups, hence the number of patients may not sum up to the total of 1,862 patients.*
