# Supplementary material for: Baseline characteristics of eyes with early residual fluid post loading phase of aflibercept therapy in neovascular AMD: PRECISE study report 3
Source: Eye (Lond). 2023 Dec 15;38(7):1301–7. doi: 10.1038/s41433-023-02886-1 (PMC11076629; doi:10.1038/s41433-023-02886-1)
Supplement: Supplementary file 3 — Table S2 [file 41433_2023_2886_MOESM3_ESM.docx]

**Table S2. Demographic and OCT features associated with early residual fluid at visit 4 - univariate and multivariable analysis using Generalised Estimating Equations (GEE) for early residual fluid, presence of eSRF and presence of eIRF**

|  | **ERF** | | | | **SRF** | | | | **IRF** | | | |
| --- | --- | --- | --- | --- | --- | --- | --- | --- | --- | --- | --- | --- |
|  | **Univariate** |  | **Multivariable** |  | **Univariate** |  | **Multivariable** |  | **Univariate** |  | **Multivariable** |  |
| **Characteristic** | **OR (95% CI)** | **p-value** | **OR (95% CI)** | **p-value** | **OR (95% CI)** | **p-value** | **OR (95% CI)** | **p-value** | **OR (95% CI)** | **p-value** | **OR (95% CI)** | **p-value** |
| **Age, per 5 year increase** | 0.83 (0.78 - 0.88) | **<0.001** |  |  | 0.79 (0.74 - 0.84) | **<0.001** |  |  | 1.04 (0.97 - 1.11) | 0.27 |  |  |
| **Age, years** |  |  |  |  |  |  |  |  |  |  |  |  |
| *< 70* | — |  | — |  | — |  | — |  | — |  | — |  |
| *70-79* | 1.02 (0.75 - 1.39) | 0.91 | 1.12 (0.81 - 1.55) | 0.50 | 0.95 (0.70 - 1.30) | 0.74 | 1.14 (0.81 - 1.60) | 0.45 | 1.29 (0.86 - 1.93) | 0.22 | 1.21 (0.77 - 1.90) | 0.40 |
| *>=80* | 0.58 (0.43 - 0.78) | **<0.001** | 0.73 (0.53 - 1.00) | **0.049** | 0.48 (0.35 - 0.65) | **<0.001** | 0.75 (0.54 - 1.05) | 0.09 | 1.32 (0.90 - 1.95) | 0.16 | 0.96 (0.62 - 1.50) | 0.86 |
| **Gender** |  |  |  |  |  |  |  |  |  |  |  |  |
| *Female* | — |  | — |  | — |  | — |  | — |  | — |  |
| *Male* | 1.33 (1.11 - 1.60) | **0.002** | 1.21 (1.00 - 1.46) | 0.06 | 1.34 (1.11 - 1.61) | **0.002** | 1.19 (0.96 - 1.47) | 0.10 | 1.13 (0.90 - 1.40) | 0.28 | 1.16 (0.91 - 1.48) | 0.23 |
| **Ethnicity** |  |  |  |  |  |  |  |  |  |  |  |  |
| *White* | — |  | — |  | — |  | — |  | — |  | — |  |
| *black/south asian/other asian/other* | 1.41 (0.93 - 2.14) | 0.11 | 1.53 (0.99 - 2.36) | 0.06 | 1.46 (0.97 - 2.22) | 0.07 | 1.62 (1.03 - 2.56) | **0.04** | 1.08 (0.66 - 1.77) | 0.77 | 1.24 (0.69 - 2.24) | 0.47 |
| **Visit 1 visual acuity categories, ETDRS** |  |  |  |  |  |  |  |  |  |  |  |  |
| *>=68* | — |  | — |  | — |  | — |  | — |  | — |  |
| *54-67* | 0.86 (0.70 - 1.07) | 0.17 | 0.88 (0.70 - 1.12) | 0.30 | 0.73 (0.58 - 0.91) | **0.004** | 0.81 (0.63 - 1.04) | 0.10 | 1.58 (1.18 - 2.12) | **0.002** | 1.20 (0.87 - 1.65) | 0.28 |
| *<54* | 0.97 (0.78 - 1.21) | 0.82 | 0.84 (0.63 - 1.12) | 0.24 | 0.65 (0.52 - 0.82) | **<0.001** | 0.70 (0.51 - 0.96) | **0.03** | 2.78 (2.10 - 3.70) | **<0.001** | 1.34 (0.93 - 1.93) | 0.11 |
| **Visit 1 visual acuity, per 5 letter increase** | 1.01 (0.98 - 1.04) | 0.71 |  |  | 1.06 (1.03 - 1.10) | **<0.001** |  |  | 0.88 (0.85 - 0.92) | **<0.001** |  |  |
| **Visit 1 visual acuity, per 5 letter decrease** | 0.99 (0.96 - 1.02) | 0.71 |  |  | 0.94 (0.91 - 0.98) | **<0.001** |  |  | 1.13 (1.09 - 1.17) | **<0.001** |  |  |
| **Central subfield thickness per 100 microns increase** | 1.26 (1.18 - 1.33) | **<0.001** | 1.31 (1.22 - 1.41) | **<0.001** | 1.20 (1.13 - 1.27) | **<0.001** | 1.29 (1.20 - 1.39) | **<0.001** | 1.23 (1.16 - 1.31) | **<0.001** | 1.19 (1.10 - 1.30) | **<0.001** |
| **Central subfield thickness quartiles** |  |  |  |  |  |  |  |  |  |  |  |  |
| *<=340* | — |  |  |  | — |  |  |  | — |  |  |  |
| *(340,415]* | 1.50 (1.17 - 1.92) | **0.001** |  |  | 1.63 (1.25 - 2.13) | **<0.001** |  |  | 1.16 (0.83 - 1.62) | 0.39 |  |  |
| *(415,525]* | 1.88 (1.46 - 2.41) | **<0.001** |  |  | 1.92 (1.48 - 2.50) | **<0.001** |  |  | 1.55 (1.13 - 2.13) | **0.007** |  |  |
| >525 | 2.66 (2.06 - 3.42) | **<0.001** |  |  | 2.29 (1.76 - 2.98) | **<0.001** |  |  | 2.55 (1.88 - 3.46) | **<0.001** |  |  |
| **CNV Type** |  |  |  |  |  |  |  |  |  |  |  |  |
| *Type 1* | — |  | — |  | — |  | — |  | — |  | — |  |
| *Type 2* | 0.86 (0.69 - 1.06) | 0.15 | 0.95 (0.72 - 1.24) | 0.69 | 0.69 (0.56 - 0.85) | **<0.001** | 0.87 (0.65 - 1.15) | 0.33 | 1.86 (1.42 - 2.44) | **<0.001** | 1.27 (0.89 - 1.81) | 0.20 |
| *RAP* | 0.41 (0.32 - 0.52) | **<0.001** | 0.70 (0.51 - 0.97) | **0.03** | 0.14 (0.10 - 0.19) | **<0.001** | 0.58 (0.38 - 0.88) | **0.01** | 2.66 (1.99 - 3.56) | **<0.001** | 1.01 (0.71 - 1.45) | 0.95 |
| *PCV* | 1.29 (0.87 - 1.90) | 0.21 | 0.98 (0.61 - 1.56) | 0.92 | 1.03 (0.70 - 1.51) | 0.88 | 0.91 (0.56 - 1.47) | 0.69 | 2.27 (1.46 - 3.53) | **<0.001** | 1.28 (0.71 - 2.28) | 0.41 |
| **Presence of any component of CNV complex** |  |  |  |  |  |  |  |  |  |  |  |  |
| *No* | — |  | — |  | — |  | — |  | — |  | — |  |
| *Yes* | 1.09 (0.63 - 1.87) | 0.77 | 0.94 (0.42 - 2.09) | 0.87 | 1.13 (0.64 - 1.99) | 0.68 | 0.70 (0.28 - 1.77) | 0.45 | 0.83 (0.44 - 1.55) | 0.55 | 0.84 (0.36 - 2.01) | 0.70 |
| **Presence of CNV** |  |  |  |  |  |  |  |  |  |  |  |  |
| *Yes, Foveal involving* | — |  | — |  | — |  | — |  | — |  | — |  |
| *Yes, Non-Foveal* | 0.93 (0.60 - 1.43) | 0.73 | 1.35 (0.70 - 2.60) | 0.37 | 0.81 (0.51 - 1.28) | 0.36 | 1.04 (0.51 - 2.13) | 0.92 | 1.16 (0.70 - 1.93) | 0.56 | 1.64 (0.80 - 3.39) | 0.18 |
| **Combination of SRF and/or IRF** |  |  |  |  |  |  |  |  |  |  |  |  |
| *IRF only* | — |  | — |  | — |  | — |  | — |  | — |  |
| *IRF and SRF* | 2.33 (1.76 - 3.08) | **<0.001** | 1.62 (1.17 - 2.22) | **0.003** | 10.3 (5.65 - 18.6) | **<0.001** | 6.79 (3.61 - 12.8) | **<0.001** | 1.71 (1.28 - 2.28) | **<0.001** | 1.31 (0.94 - 1.83) | 0.11 |
| *SRF only* | 3.57 (2.73 - 4.67) | **<0.001** | 2.26 (1.59 - 3.20) | **<0.001** | 35.1 (19.5 - 63.0) | **<0.001** | 19.9 (10.4 - 37.9) | **<0.001** | 0.24 (0.17 - 0.34) | **<0.001** | 0.22 (0.14 - 0.33) | **<0.001** |
| **Presence of Pigment Epithelial Detachment** |  |  |  |  |  |  |  |  |  |  |  |  |
| *No* | — |  | — |  | — |  | — |  | — |  | — |  |
| *Yes, Foveal involving* | 1.13 (0.76 - 1.68) | 0.55 | 1.02 (0.66 - 1.56) | 0.94 | 1.42 (0.93 - 2.19) | 0.11 | 1.14 (0.71 - 1.85) | 0.58 | 0.81 (0.51 - 1.30) | 0.38 | 1.13 (0.68 - 1.87) | 0.64 |
| *Yes, Non-Foveal* | 1.08 (0.69 - 1.68) | 0.74 | 1.38 (0.86 - 2.21) | 0.19 | 1.16 (0.72 - 1.86) | 0.54 | 1.47 (0.86 - 2.52) | 0.16 | 0.92 (0.54 - 1.55) | 0.75 | 1.31 (0.74 - 2.31) | 0.36 |
| **Presence of Atrophy or outer retinal tubulation** | 0.54 (0.43 - 0.66) | **<0.001** | 0.70 (0.50 - 0.99) | **0.04** | 0.34 (0.26 - 0.44) | **<0.001** | 0.63 (0.42 - 0.93) | **0.02** | 1.41 (1.11 - 1.80) | **0.006** | 0.78 (0.53 - 1.15) | 0.20 |
| **Presence of fibrosis** |  |  |  |  |  |  |  |  |  |  |  |  |
| *No* | — |  | — |  | — |  | — |  | — |  | — |  |
| *Yes* | 1.33 (1.04 - 1.71) | **0.02** | 1.33 (0.92 - 1.92) | 0.13 | 0.90 (0.70 - 1.17) | 0.43 | 1.31 (0.85 - 2.01) | 0.22 | 2.55 (1.95 - 3.33) | **<0.001** | 1.23 (0.82 - 1.84) | 0.33 |
| **Presence of subretinal hyperreflective material** |  |  |  |  |  |  |  |  |  |  |  |  |
| *No* | — |  | — |  | — |  | — |  | — |  | — |  |
| *Yes* | 1.04 (0.87 - 1.25) | 0.65 | 0.93 (0.71 - 1.22) | 0.60 | 1.06 (0.88 - 1.28) | 0.52 | 0.93 (0.70 - 1.24) | 0.63 | 1.25 (1.00 - 1.55) | 0.05 | 0.92 (0.64 - 1.30) | 0.62 |
| **Presence of Drusen** |  |  |  |  |  |  |  |  |  |  |  |  |
| *No* | — |  | — |  | — |  | — |  | — |  | — |  |
| *Yes* | 0.90 (0.65 - 1.24) | 0.52 | 1.13 (0.76 - 1.66) | 0.55 | 1.02 (0.73 - 1.43) | 0.89 | 1.30 (0.84 - 2.00) | 0.24 | 0.76 (0.53 - 1.09) | 0.14 | 0.99 (0.62 - 1.58) | 0.96 |
| **Presence of subretinal drusenoid deposit** |  |  |  |  |  |  |  |  |  |  |  |  |
| *No* | — |  | — |  | — |  | — |  | — |  | — |  |
| *Yes* | 0.70 (0.58 - 0.85) | **<0.001** | 1.04 (0.83 - 1.29) | 0.75 | 0.57 (0.46 - 0.70) | **<0.001** | 1.00 (0.78 - 1.29) | 0.99 | 1.06 (0.84 - 1.35) | 0.60 | 1.01 (0.77 - 1.33) | 0.96 |
| **Hyperreflective Foci** |  |  |  |  |  |  |  |  |  |  |  |  |
| *No* | — |  | — |  | — |  | — |  | — |  | — |  |
| *Yes* | 1.03 (0.85 - 1.25) | 0.74 | 1.16 (0.94 - 1.43) | 0.17 | 0.95 (0.78 - 1.16) | 0.63 | 1.19 (0.94 - 1.51) | 0.14 | 1.31 (1.03 - 1.67) | **0.03** | 1.03 (0.78 - 1.35) | 0.85 |
| **Presence of VMT or ERM** |  |  |  |  |  |  |  |  |  |  |  |  |
| *No* | — |  | — |  | — |  | — |  | — |  | — |  |
| *Yes* | 1.00 (0.77 - 1.31) | 0.99 | 1.19 (0.89 - 1.59) | 0.25 | 0.64 (0.48 - 0.86) | **0.003** | 0.78 (0.56 - 1.10) | 0.15 | 1.65 (1.22 - 2.22) | **0.001** | 1.61 (1.14 - 2.26) | **0.006** |
| **EZ/ELM** |  |  |  |  |  |  |  |  |  |  |  |  |
| *Neither EZ/ELM loss* | — |  | — |  | — |  | — |  | — |  | — |  |
| *EZ/ELM loss* | 0.69 (0.56 - 0.86) | **<0.001** | 0.92 (0.62 - 1.35) | 0.66 | 0.42 (0.33 - 0.52) | **<0.001** | 0.84 (0.54 - 1.31) | 0.45 | 2.99 (2.28 - 3.90) | **<0.001** | 1.54 (0.96 - 2.45) | 0.07 |
| *Both ungradable* | 0.68 (0.55 - 0.84) | **<0.001** | 0.70 (0.53 - 0.92) | **0.01** | 0.58 (0.47 - 0.73) | **<0.001** | 0.79 (0.59 - 1.07) | 0.13 | 2.03 (1.53 - 2.68) | **<0.001** | 1.12 (0.77 - 1.65) | 0.56 |

Abbreviations: OCT- Optical coherence tomography; ETDRS- Early Treatment Diabetic Retinopathy Study; GEE -Generalised Estimating Equation; VA- Visual Acuity; OR- Odds Ratio; CI- Confidence interval

^a^ Not included in multivariable analysis
