# Supplementary material for: Baseline characteristics of eyes with early residual fluid post loading phase of aflibercept therapy in neovascular AMD: PRECISE study report 3
Source: Eye (Lond). 2023 Dec 15;38(7):1301–7. doi: 10.1038/s41433-023-02886-1 (PMC11076629; doi:10.1038/s41433-023-02886-1)
Supplement: Supplementary file 4 — Table S3 [file 41433_2023_2886_MOESM4_ESM.docx]

**Table S3. Sensitivity analysis on eyes that were observed during the follow-up window (8 +/- 2 weeks from the last injection or at least 104 days from the first aflibercept injection) - univariate and multivariable analysis using Generalised Estimating Equations (GEE) for early residual fluid, presence of eSRF and presence of eIRF**

|  | **ERF** | | | | **SRF** | | | | **IRF** | | | |
| --- | --- | --- | --- | --- | --- | --- | --- | --- | --- | --- | --- | --- |
|  | **Univariate** | | **Multivariable** | | **Univariate** | | **Multivariable** | | **Univariate** | | **Multivariable** | |
| **Characteristic** | **OR** | **p-value** | **OR** | **p-value** | **OR** | **p-value** | **OR** | **p-value** | **OR** | **p-value** | **OR** | **p-value** |
| **Age, per 5 year increase** | 0.79 (0.73 - 0.85) | **<0.001** | NA^a^ | NA^a^ | 0.74 (0.69 - 0.80) | **<0.001** | NA^a^ | NA^a^ | 1.04 (0.96 - 1.12) | 0.32 | NA^a^ | NA^a^ |
| **Age, years** |  |  |  |  |  |  |  |  |  |  |  |  |
| *< 70* | — |  | — |  | — |  | — |  | — |  | — |  |
| *70-79* | 0.93 (0.63 - 1.37) | 0.71 | 1.05 (0.70 - 1.58) | 0.82 | 0.89 (0.61 - 1.29) | 0.53 | 1.08 (0.71 - 1.63) | 0.73 | 1.27 (0.80 - 2.02) | 0.31 | 1.30 (0.77 - 2.20) | 0.33 |
| *>=80* | 0.48 (0.33 - 0.69) | **<0.001** | 0.62 (0.41 - 0.92) | **0.02** | 0.40 (0.28 - 0.57) | **<0.001** | 0.61 (0.40 - 0.91) | **0.02** | 1.36 (0.87 - 2.13) | 0.17 | 1.10 (0.65 - 1.85) | 0.73 |
| **Gender** |  |  |  |  |  |  |  |  |  |  |  |  |
| *Female* | — |  | — |  | — |  | — |  | — |  | — |  |
| *Male* | 1.36 (1.09 - 1.69) | **0.007** | 1.25 (0.98 - 1.58) | 0.07 | 1.34 (1.08 - 1.67) | **0.009** | 1.25 (0.97 - 1.62) | 0.09 | 1.19 (0.93 - 1.53) | 0.17 | 1.24 (0.94 - 1.65) | 0.13 |
| **Ethnicity** |  |  |  |  |  |  |  |  |  |  |  |  |
| *White* | — |  | — |  | — |  | — |  | — |  | — |  |
| *black/south asian/other asian/other* | 1.40 (0.83 - 2.37) | 0.21 | 1.57 (0.90 - 2.74) | 0.12 | 1.44 (0.87 - 2.38) | 0.16 | 1.69 (0.96 - 2.97) | 0.07 | 1.13 (0.64 - 1.99) | 0.67 | 1.36 (0.69 - 2.72) | 0.38 |
| **Visit 1 visual acuity categories, ETDRS** |  |  |  |  |  |  |  |  |  |  |  |  |
| *>=68* | — |  | — |  | — |  | — |  | — |  | — |  |
| *54-67* | 0.78 (0.61 - 1.01) | 0.058 | 0.85 (0.64 - 1.14) | 0.28 | 0.69 (0.53 - 0.89) | **0.004** | 0.87 (0.64 - 1.19) | 0.38 | 1.50 (1.07 - 2.09) | **0.017** | 1.04 (0.72 - 1.51) | 0.83 |
| *<54* | 0.94 (0.72 - 1.22) | 0.64 | 0.99 (0.70 - 1.41) | 0.96 | 0.61 (0.47 - 0.80) | **<0.001** | 0.86 (0.58 - 1.26) | 0.44 | 2.99 (2.16 - 4.13) | **<0.001** | 1.37 (0.90 - 2.10) | 0.14 |
| **Visit 1 visual acuity, per 5 letter increase** | 1.01 (0.98 - 1.05) | 0.46 | NA^a^ | NA^a^ | 1.07 (1.03 - 1.12) | **<0.001** | NA^a^ | NA^a^ | 0.87 (0.84 - 0.91) | **<0.001** | NA^a^ | NA^a^ |
| **Visit 1 visual acuity, per 5 letter decrease** | 0.99 (0.95 - 1.02) | 0.46 | NA^a^ | NA^a^ | 0.93 (0.89 - 0.97) | **<0.001** | NA^a^ | NA^a^ | 1.14 (1.10 - 1.20) | **<0.001** | NA^a^ | NA^a^ |
| **Central subfield thickness per 100 microns increase** | 1.26 (1.17 - 1.35) | **<0.001** | 1.30 (1.19 - 1.42) | **<0.001** | 1.18 (1.10 - 1.26) | **<0.001** | 1.24 (1.13 - 1.37) | **<0.001** | 1.25 (1.17 - 1.34) | **<0.001** | 1.26 (1.15 - 1.38) | **<0.001** |
| **Central subfield thickness quartiles, microns** |  |  |  |  |  |  |  |  |  |  |  |  |
| <=340 | — |  |  |  | — |  |  |  | — |  |  |  |
| *(340,415]* | 1.80 (1.34 - 2.43) | **<0.001** | NA^a^ | NA^a^ | 1.91 (1.40 - 2.60) | **<0.001** | NA^a^ | NA^a^ | 1.20 (0.82 - 1.77) | 0.35 | NA^a^ | NA^a^ |
| *(415,525]* | 1.87 (1.39 - 2.52) | **<0.001** | NA^a^ | NA^a^ | 1.87 (1.38 - 2.54) | **<0.001** | NA^a^ | NA^a^ | 1.53 (1.06 - 2.20) | **0.023** | NA^a^ | NA^a^ |
| >525 | 2.89 (2.14 - 3.92) | **<0.001** | NA^a^ | NA^a^ | 2.23 (1.65 - 3.02) | **<0.001** | NA^a^ | NA^a^ | 2.69 (1.90 - 3.82) | **<0.001** | NA^a^ | NA^a^ |
| **CNV Type** |  |  |  |  |  |  |  |  |  |  |  |  |
| *Type 1* | — |  | — |  | — |  | — |  | — |  | — |  |
| *Type 2* | 0.77 (0.60 - 1.00) | 0.051 | 0.79 (0.56 - 1.13) | 0.20 | 0.63 (0.49 - 0.81) | **<0.001** | 0.76 (0.53 - 1.10) | 0.15 | 1.92 (1.41 - 2.62) | **<0.001** | 1.22 (0.79 - 1.88) | 0.38 |
| *RAP* | 0.35 (0.26 - 0.47) | **<0.001** | 0.65 (0.44 - 0.97) | **0.04** | 0.11 (0.08 - 0.16) | **<0.001** | 0.46 (0.28 - 0.75) | **0.002** | 2.91 (2.08 - 4.07) | **<0.001** | 1.06 (0.69 - 1.65) | 0.78 |
| *PCV* | 0.95 (0.60 - 1.52) | 0.84 | 0.67 (0.38 - 1.18) | 0.16 | 0.79 (0.51 - 1.24) | 0.31 | 0.62 (0.35 - 1.12) | 0.11 | 2.11 (1.26 - 3.51) | **0.004** | 1.08 (0.57 - 2.07) | 0.81 |
| **Presence of any component of CNV complex** |  |  |  |  |  |  |  |  |  |  |  |  |
| *No* | — |  | — |  | — |  | — |  | — |  | — |  |
| *Yes* | 1.25 (0.69 - 2.27) | 0.46 | 1.07 (0.39 - 2.91) | 0.89 | 1.40 (0.75 - 2.61) | 0.29 | 0.95 (0.31 - 2.88) | 0.92 | 0.77 (0.40 - 1.49) | 0.44 | 0.68 (0.26 - 1.82) | 0.45 |
| **Presence of CNV** |  |  |  |  |  |  |  |  |  |  |  |  |
| *Yes, Foveal involving* | — |  | — |  | — |  | — |  | — |  | — |  |
| *Yes, Non-Foveal* | 0.88 (0.55 - 1.43) | 0.61 | 1.58 (0.70 - 3.57) | 0.28 | 0.75 (0.46 - 1.23) | 0.26 | 1.27 (0.52 - 3.07) | 0.60 | 1.16 (0.67 - 1.98) | 0.60 | 1.56 (0.66 - 3.65) | 0.31 |
| **Combination of SRF and/or IRF** |  |  |  |  |  |  |  |  |  |  |  |  |
| *IRF only* | — |  | — |  | — |  | — |  | — |  | — |  |
| *IRF and SRF* | 2.35 (1.70 - 3.27) | **<0.001** | 1.67 (1.15 - 2.43) | **0.007** | 11.0 (5.65 - 21.3) | **<0.001** | 7.50 (3.76 - 15.0) | **<0.001** | 1.72 (1.24 - 2.40) | **0.001** | 1.32 (0.89 - 1.95) | 0.16 |
| *SRF only* | 4.27 (3.12 - 5.85) | **<0.001** | 2.48 (1.64 - 3.76) | **<0.001** | 43.2 (22.5 - 83.1) | **<0.001** | 21.9 (10.9 - 44.0) | **<0.001** | 0.24 (0.16 - 0.35) | **<0.001** | 0.20 (0.12 - 0.32) | **<0.001** |
| **Presence of Pigment Epithelial Detachment** |  |  |  |  |  |  |  |  |  |  |  |  |
| *No* | — |  | — |  | — |  | — |  | — |  | — |  |
| *Yes, Foveal involving* | 1.35 (0.85 - 2.15) | 0.20 | 1.11 (0.68 - 1.82) | 0.67 | 1.87 (1.14 - 3.07) | **0.01** | 1.49 (0.87 - 2.56) | 0.14 | 0.73 (0.44 - 1.22) | 0.24 | 1.01 (0.58 - 1.74) | 0.98 |
| *Yes, Non-Foveal* | 1.08 (0.65 - 1.79) | 0.77 | 1.35 (0.79 - 2.30) | 0.28 | 1.26 (0.74 - 2.16) | 0.40 | 1.62 (0.90 - 2.91) | 0.11 | 0.80 (0.46 - 1.40) | 0.43 | 1.21 (0.66 - 2.21) | 0.55 |
| **Presence of Atrophy or outer retinal tubulation** | 0.47 (0.37 - 0.60) | **<0.001** | 0.67 (0.46 - 0.99) | **0.045** | 0.31 (0.23 - 0.41) | **<0.001** | 0.63 (0.41 - 0.96) | **0.03** | 1.35 (1.03 - 1.78) | **0.03** | 0.68 (0.44 - 1.05) | 0.08 |
| **Presence of fibrosis** |  |  |  |  |  |  |  |  |  |  |  |  |
| *No* | — |  | — |  | — |  | — |  | — |  | — |  |
| *Yes* | 1.17 (0.88 - 1.57) | 0.27 | 1.15 (0.75 - 1.77) | 0.52 | 0.83 (0.62 - 1.12) | 0.22 | 1.25 (0.77 - 2.03) | 0.38 | 2.35 (1.74 - 3.18) | **<0.001** | 0.97 (0.61 - 1.56) | 0.91 |
| **Presence of subretinal hyperreflective material** |  |  |  |  |  |  |  |  |  |  |  |  |
| *No* | — |  | — |  | — |  | — |  | — |  | — |  |
| *Yes* | 1.11 (0.89 - 1.37) | 0.35 | 1.15 (0.81 - 1.65) | 0.43 | 1.10 (0.89 - 1.37) | 0.37 | 1.07 (0.73 - 1.55) | 0.74 | 1.30 (1.01 - 1.67) | **0.04** | 1.04 (0.69 - 1.58) | 0.84 |
| **Presence of Drusen** |  |  |  |  |  |  |  |  |  |  |  |  |
| *No* | — |  | — |  | — |  | — |  | — |  | — |  |
| *Yes* | 0.93 (0.64 - 1.36) | 0.70 | 1.01 (0.65 - 1.59) | 0.96 | 1.00 (0.69 - 1.46) | 0.99 | 1.07 (0.65 - 1.77) | 0.79 | 0.77 (0.51 - 1.16) | 0.21 | 0.94 (0.57 - 1.57) | 0.82 |
| **Presence of subretinal drusenoid deposit** |  |  |  |  |  |  |  |  |  |  |  |  |
| *No* | — |  | — |  | — |  | — |  | — |  | — |  |
| *Yes* | 0.73 (0.58 - 0.92) | **0.007** | 1.10 (0.84 - 1.44) | 0.49 | 0.64 (0.50 - 0.80) | **<0.001** | 1.20 (0.88 - 1.62) | 0.24 | 1.03 (0.79 - 1.34) | 0.83 | 0.93 (0.68 - 1.28) | 0.67 |
| **Hyperreflective Foci** |  |  |  |  |  |  |  |  |  |  |  |  |
| *No* | — |  | — |  | — |  | — |  | — |  | — |  |
| *Yes* | 0.93 (0.73 - 1.18) | 0.54 | 1.08 (0.83 - 1.42) | 0.56 | 0.89 (0.70 - 1.13) | 0.34 | 1.21 (0.90 - 1.62) | 0.20 | 1.18 (0.89 - 1.58) | 0.25 | 0.86 (0.62 - 1.18) | 0.35 |
| **Presence of VMT or ERM** |  |  |  |  |  |  |  |  |  |  |  |  |
| *No* | — |  | — |  | — |  | — |  | — |  | — |  |
| *Yes* | 1.06 (0.76 - 1.49) | 0.72 | 1.35 (0.93 - 1.97) | 0.11 | 0.71 (0.50 - 1.00) | 0.051 | 0.92 (0.60 - 1.41) | 0.71 | 1.61 (1.13 - 2.30) | **0.009** | 1.69 (1.11 - 2.56) | **0.01** |
| **EZ/ELM** |  |  |  |  |  |  |  |  |  |  |  |  |
| *Neither EZ/ELM loss* | — |  | — |  | — |  | — |  | — |  | — |  |
| *EZ/ELM loss* | 0.56 (0.44 - 0.72) | **<0.001** | 0.80 (0.51 - 1.27) | 0.35 | 0.34 (0.27 - 0.45) | **<0.001** | 0.70 (0.43 - 1.16) | 0.17 | 3.01 (2.21 - 4.10) | **<0.001** | 1.51 (0.89 - 2.56) | 0.13 |
| *Both ungradable* | 0.56 (0.43 - 0.73) | **<0.001** | 0.59 (0.42 - 0.83) | **0.003** | 0.49 (0.38 - 0.63) | **<0.001** | 0.70 (0.48 - 1.01) | 0.06 | 1.99 (1.44 - 2.76) | **<0.001** | 0.95 (0.60 - 1.49) | 0.81 |

Abbreviations: OCT- Optical coherence tomography; ETDRS- Early Treatment Diabetic Retinopathy Study; GEE -Generalised Estimating Equation; VA- Visual Acuity; OR- Odds Ratio; CI- Confidence interval

^a^ Not included in multivariable analysis
